# Noninvasive prediction of CCL2 expression level in high‐grade glioma patients

**DOI:** 10.1002/cam4.70016

**Published:** 2024-07-18

**Authors:** Qingqing Zhou, Yamei Wang, Qing Zhang, XiaoMing Wei, Yuan Yao, Liang Xia

**Affiliations:** ^1^ Department of Neurosurgery The First Affiliated Hospital of Yangtze University, Jingzhou First People's Hospital Jingzhou People's Republic of China; ^2^ Department of Neurology The First Affiliated Hospital of Yangtze University, Jingzhou First People's Hospital Jingzhou People's Republic of China; ^3^ Department of Radiology The First Affiliated Hospital of Yangtze University, Jingzhou First People's Hospital Jingzhou People's Republic of China; ^4^ Department of Neurosurgery The Cancer Hospital of the University of Chinese Academy of Sciences (Zhejiang Cancer Hospital), Institute of Basic Medicine and Cancer (IBMC), Chinese Academy of Sciences Hangzhou People's Republic of China

**Keywords:** CCL2 expression level, gliomas, machine learning, MRI‐based radiomics, noninvasive prediction

## Abstract

**Background:**

Gliomas are recognized as the most frequent type of malignancies in the central nervous system, and efficacious prognostic indicators are essential to treat patients with gliomas and improve their clinical outcomes. The chemokine (C‐C motif) ligand 2 (CCL2) is a promising predictor for glioma malignancy and progression. However, at present, the methods to evaluate CCL2 expression level are invasive and operator‐dependent.

**Objective:**

It was expected to noninvasively predict CCL2 expression levels in malignant glioma tissues by magnetic resonance imaging (MRI)‐based radiomics and assess the association between the developed radiomics model and prognostic indicators and related genes.

**Methods:**

MRI‐based radiomics was used to predict CCL2 expression level using data obtained from The Cancer Imaging Archive (TCIA) and The Cancer Genome Atlas (TCGA) databases. A support vector machine (SVM)‐based radiomics model and a logistic regression (LR)‐based radiomics model were used to predict the radiomics score, and its correlation with CCL2 expression level was analyzed.

**Results:**

The results revealed that there was an association between CCL2 expression level and the overall survival of cases with gliomas, and bioinformatics correlation analysis showed that CCL2 expression level was highly correlated with disease‐related pathways, such as mTOR signaling pathway, cGMP‐PKG signaling pathway, and MAPK signaling pathway. Both SVM‐ and LR‐based radiomics data robustly predicted CCL2 expression level, and radiomics scores could also be used to predict the overall survival of patients. Moreover, the high/low radiomics scores were highly correlated with the known glioma‐related genes, including CD70, CD27, and PDCD1.

**Conclusion:**

An MRI‐based radiomics model was successfully developed, and its clinical benefits were confirmed, including the prediction of CCL2 expression level and patients' prognosis.

## 1 INTRODUCTION

Gliomas represent the most prevalent form of malignancy within the central nervous system (CNS), constituting 40%–50% of CNS tumors and approximately 80% of CNS malignancies.^1^, ^2^ Despite the availability of various therapeutic modalities for gliomas, including chemotherapy, surgery, targeted therapy, and radiotherapy, patients with glioma continue to have a dismal prognosis or outcome. The World Health Organization (WHO) classification system categorizes gliomas into low‐grade (I–II) and high‐grade (III–IV) tumors, with low‐grade gliomas typically associated with a more favorable prognosis compared with high‐grade gliomas (HGGs), exhibiting a higher degree of malignancy and a poorer prognosis.^2^ Therefore, a robust predictive model is vital for clinical diagnosis, treatment plans, and prognostic judgment. At present, prognostic indicators of gliomas include clinicopathological features, such as Ki67,^3^, ^4^ IDH,^5^, ^6^, ^7^ MGMT promoter methylation (MGMT‐PM),^8^, ^9^ and 1p/19q co‐deletion.^7^, ^10^, ^11^ However, these indicators are invasive. Therefore, it is necessary to further explore new noninvasive prognostic markers to stratify the prognosis of patients and develop novel precise treatment plans.

Two adjacent cysteine residues characterize chemokine (C‐C motif) ligand 2 (CCL2), a member of the CC subfamily of cytokines. Known for its chemotactic activity targeting monocytes and basophils rather than neutrophils or eosinophils, CCL2 is implicated in the pathogenesis of chronic inflammatory diseases, such as psoriasis,^12^, ^13^ rheumatoid arthritis,^14^, ^15^ and atherosclerosis.^16^, ^17^ It binds to the chemokine receptors CCR2 and CCR4, and it acts as one of the key chemokine pathways for glioblastoma multiform^18^ and malignancy.^19^ Shono et al. found that in a mouse model of malignant gliomas, the downregulation of CCL2/CCR2 was resulted in the reduction in glioma stem cell viability.^20^ In addition, a recent clinical trial used CCL2 expression level as a secondary measure of efficacy for glioblastoma patients' treatment using Salovum egg yolk powder (ClinicalTrials.gov, Identifier: NCT04116138).

While the prognostic value of CCL2 expression level in gliomas has been documented, its detection presents challenges through various pathways: (1) peripheral blood cytokine detection provides real‐time assessment, while it may be costly and fail to reflect the expression of CCL2 in the tumor parenchyma^21^, ^22^; (2) mRNA (quantitative polymerase chain reaction (qPCR), RNA‐seq) or protein level tests (Western blotting, flow cytometry, etc.) based on fresh tissue samples are hindered by specimen collection difficulties, operator‐dependent testing, and susceptibility to antibody effects^23^, ^24^; (3) The detection of paraffin‐embedded tissue samples by immunohistochemistry, fluorescence, or high‐throughput sequencing may also exhibit defects, such as being operator‐dependent, the usage of antibodies, and being costly.^25^, ^26^ Although previous studies showed the correlation of CCL2/CCR2 with glioma recurrence and poor survival rates in preclinical^27^ and clinical^28^ settings, along with their relevance to MRI diagnostic results, no significant correlation between CCL2 expression level and MRI data have been proposed.

The radiomics quantitatively analyzes images to assess tumors noninvasively,^29^, ^30^, ^31^ and it can be well‐suited to predict the CCL2 expression level in glioma patients. This study aimed to innovatively and noninvasively predict CCL2 expression level in HGG patients by MRI‐based radiomics and assess the association between the developed radiomics model and prognostic indicators and the associated genes. Moreover, the molecular mechanism underlying CCL2 expression level and its association with the immune microenvironment were explored and validated using bioinformatics analysis.

## 2 MATERIALS AND METHODS

### 2.1 Patients' data and classification

Transcriptome sequencing data and medical imaging data (e.g., follow‐up and clinical data) were obtained from The Cancer Genome Atlas (TCGA) and The Cancer Imaging Archive (TCIA) databases to assess the prognostic value of CCL2 expression level and the MRI‐based radiomics predictive model. Among 310 cases with HGGs from TCGA database included in the survival analysis, the cutoff value of CCL2 expression level equal to 3.31465 was obtained using the “survminer” R package, which is an extension of the “survival” package, to perform survival analysis and visualize Kaplan–Meier survival curves. The “survminer” package leverages the plotting capabilities of “ggplot2,” a widely used R package for creating elegant and customizable graphics. Thereafter, cases were classified into low‐expression group (*n* = 134) and high‐expression group (*n* = 176). The summary of cases' clinical data is presented in Table 1. For the MRI‐based radiomics analysis, the bioinformatics data from TCGA and imaging data from TCIA were intersected, and the number of intersecting samples was equal to 86. Besides, 107 standardized radiomics features were extracted by the “pyradiomics” R package.

**TABLE 1:** Summary of the clinical data of the high CCL2 expression and the low CCL2 expression groups.

Variables	Total (*n* = 310)	Low (*n* = 134)	High (*n* = 176)	*p*‐value
Age, *n* (%)				<0.001
<59	208 (67)	111 (83)	97 (55)	
>60	102 (33)	23 (17)	79 (45)	
Gender, *n* (%)				0.048
Male	125 (40)	63 (47)	62 (35)	
Female	185 (60)	71 (53)	114 (65)	
Radiotherapy, *n* (%)				0.128
Yes	65 (21)	34 (25)	50 (28)	
No	245 (79)	100 (75)	126 (72)	
Chemotherapy, *n* (%)				0.09
Yes	76 (25)	26 (19)	31 (18)	
No	234 (75)	108 (81)	145 (82)	
IDH_status, *n* (%)				<0.001
Wild‐type	171 (55)	38 (28)	133 (76)	
Mutant	139 (45)	96 (72)	43 (24)	

### 2.2 Enrichment analysis

The enrichment analysis was conducted to explore the correlation of the CCL2 expression level with the genome‐based differences. The Gene Ontology (GO) is widely used to annotate genes with functions, particularly cellular component (CC), biological process (BP), and molecular function (MF). The Kyoto Encyclopedia of Genes and Genomes (KEGG) database stores information related to drugs, biological pathways, genomes, and diseases. In this study, “clusterProfiler” R package was utilized for and KEGG pathway and GO (BP/CC/MF) enrichment analyses with *q*valueFilter <0.05 as the screening criterion. The top 10 significantly enriched terms were visualized for the GO enrichment analysis, and the top 30 enriched pathways were visualized for the KEGG pathway enrichment analysis.

### 2.3 Volume‐of‐interest segmentation and extraction of radiomics features

Volume of interests (VOIs) was delineated by two oncologists with 3D slicer, who marked the outline of the tumor to highlight the full tumor area. Once one oncologist completed extraction of all features, 30 samples were selected randomly using the “random number table method” to be sketched by another oncologist.

### 2.4 Feature selection

It was attempted to indicate the consistency of extracted radiomics features via intraclass correlation coefficient (ICC). It is generally accepted that ICC ≥0.8, equal to 0.51–0.79, and <0.50 indicate high consistency, medium consistency, and poor consistency, respectively. The median ICC value of radiomics features was 0.95, and ICC value ≥0.8 was found in 101 (94.4%) out of 107 radiomics features.^32^

The maximal relevance and minimal redundancy (mRMR) method was used to screen features. The method not only considers the correlation between the feature and the variable for prediction, but also assesses the correlation between features and features. Mutual information was used as the metric. For the mRMR method, the average of the information gain of each feature and class was utilized for the purpose of calculating correlation of a subset of features to a class, while the sum of mutual information between features that was divided by the square of the number of features in the subset was used for calculating redundancy.

Besides, recursive feature elimination (RFE) was used for feature screening. Before modeling, predictors were sorted, and unimportant factors were eliminated with the given order.^33^ Exploration of a subset of predictors to develop accurate models was the main objective. With the continuous training of the model, *n* features of low importance were deleted, and the features after deletion were then re‐trained. The best subset of features was obtained.

### 2.5 Development and evaluation of the SVM‐based radiomics models

The support vector machine (SVM) algorithm was used for the development of an SVM‐based radiomics model. In the SVM, the hyperplane at the high latitude was identified by the support vectors as the decision boundary. Then, five radiomics signatures screened above were modeled by the SVM algorithm using the “caret” R package for gene expression prediction.

Based on linear regression, logistic regression (LR) includes the sigmoid function, which is widely used in the binary classification of problems, and it is formulated as follows: $S\left(x\right)=\frac{1}{1+e^{-x}}S\left(x\right)=\frac{1}{1+e^{-x}}$. The “stats” R package was used to fit the logistic regression algorithm of the five radiomics features, in order to establish a dichotomous model for predicting gene expression. Radiomics was formulated as follows: radiomics = Feature * the factor corresponding to the feature (Estimate) + Intercept value (Estimate). Similar to the evaluation of the SVM‐based radiomics model, indicators, including ACC, SPE, SEN, PPV, and NPV were used. ROC, PR, and DCA curves were plotted for assessment of the performance of the LR‐based radiomics model.

### 2.6 Statistical analysis

To evaluate model performance, indicators (e.g., negative predictive value [NPV], positive predictive value [PPV], sensitivity [SEN], specificity [SPE], and accuracy [ACC]) were utilized. The false‐positive rate (FPR) and true‐positive rate (TPR) were labeled in the *X*‐ and *Y*‐axis of the receiver operating characteristic (ROC) curve. The larger the area under the curve (AUC), the more convex the curve in the upper left corner, and the better performance of the model. The recall and precision were labeled in the *X*‐ and *Y*‐axis of the precision–recall (PR) curve. The mean accuracy was denoted by PR‐AUC for each coverage threshold. The more the PR curve is convex to the upper right corner, the better the model performance. It was attempted to assess calibration of the radiomics prediction model through drawing the calibration curve, followed by implementation of the Hosmer–Lemeshow goodness‐of‐fit (HLGOF) test. The Brier score was calculated for assessing the performance of the prediction model. The smaller the Brier score, the better the consistency of the model prediction. The clinical benefits of the radiomics prediction model were demonstrated using the decision curve analysis (DCA). To compare training and validation datasets in terms of AUC values, the DeLong test was used.

The CCL2 expression levels were analyzed by processing the RNA‐seq data in the format of transcripts per million (TPM) reads from TCGA and GTEx databases using Toil process.^33^ GBM/LGG (glioma) data were extracted from TCGA database, and the normal tissue data were extracted from the GTEx database. Then, log2 conversion of RNAseq data in TPM format was performed for differential expression analysis between samples. Differences in the degree of immune cell infiltration between the high CCL2 expression and low CCL2 expression groups were analyzed by the “limma” R package. Each variable was analyzed separately using the “survival” “forestplot” R packages, and the outputs were used to summarize and visualize the results of the analysis. Differences in Rad_score between the high and low CCL2 expression groups were compared by Wilcoxon test. The correlation between the host variable (CCL2 expression level) and the clinical features of patients with gliomas was analyzed using the Spearman's correlation coefficient.

## 3 RESULTS

### 3.1 Correlation between patients' characteristics and CCL2 expression level

**FIGURE 1:**
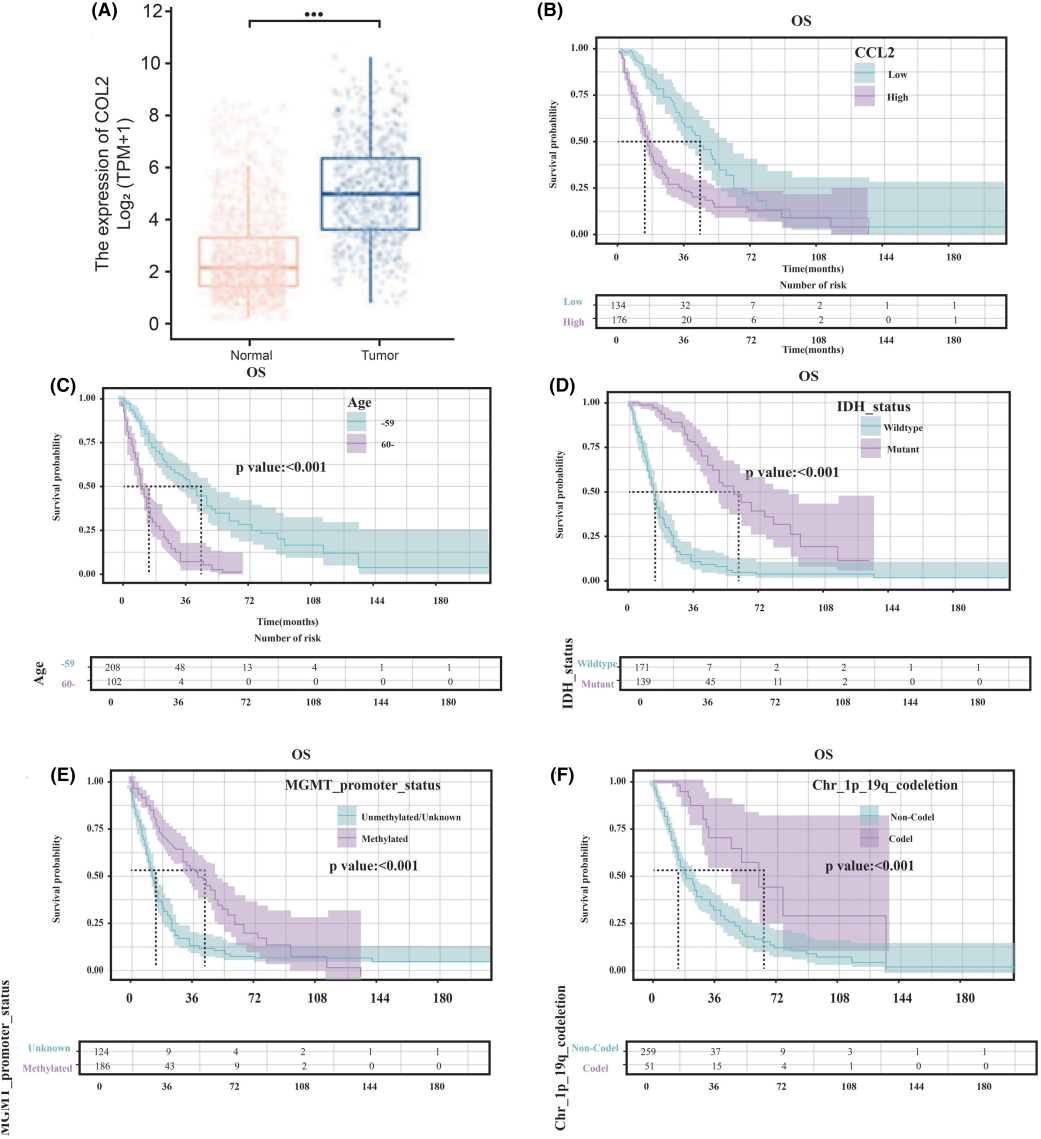
(A) Comparison of CCL2 expression level between normal and tumor groups. KMSC for different risk factors: (B) CCL2 expression level, (C) Patients' age, (D) IDH status, (E) MGMT‐PM status, and (F) Co‐deletion status of Chr 1p/19q.

As shown in Figure 1A, the tumor group exhibited a higher CCL2 expression level than that in the normal group, indicating a median difference of 2.653 between the two groups (2.478–2.829, *p* < 0.001). Next, comparison of survival rates among different groups was undertaken by the Kaplan–Meier survival curve (KMSC), and the significance of survival was investigated between groups via the log‐rank test. As illustrated in Figure 1B, the median survival time was 44.63 months in the low CCL2 expression group, while it was only 16.83 months in the high CCL2 expression group. The difference in median survival times between groups with low and high CCL2 expression levels could highlight the potential significance of CCL2 in determining prognosis, reflecting its role in immune response and inflammation. The notably longer median survival time in the low CCL2 expression group suggested a potential protective effect associated with the lower CCL2 level, indicating a less aggressive disease phenotype. The KMSC showed that the higher CCL2 expression level was significantly associated with the reduction of the overall survival (OS) rate (*p* < 0.001). Other parameters were also analyzed. The median survival time was 41.1 months in the group of 59 years old or younger, while the median survival time in the group of 60 years old or older was only 13.8 months. The KMSC (Figure 1C) showed that age older than or equal to 60 years was significantly associated with the reduction in OS rate (*p* < 0.001). Other important factors, including isocitrate dehydrogenase (IDH) enzymes, that were previously found as mutated in gliomas,^34^ and biomarkers (e.g., methylation of the O^6^‐methylguanine‐DNA methyltransferase (MGMT) promoter), were found to be associated with the silencing of MGMT as a favorable outcome.^8^ Moreover, Chr 1p/19q co‐deletion, as an indicator of the patient survival,^35^ was significantly correlated with the KMSC (Figure 1D–F). These results demonstrated that CCL2 was highly expressed and its role as a significant risk factor for OS rate, especially in patients with gliomas, was confirmed.

### 3.2 Survival analysis of the risk factors using COX regression model

**FIGURE 2:**
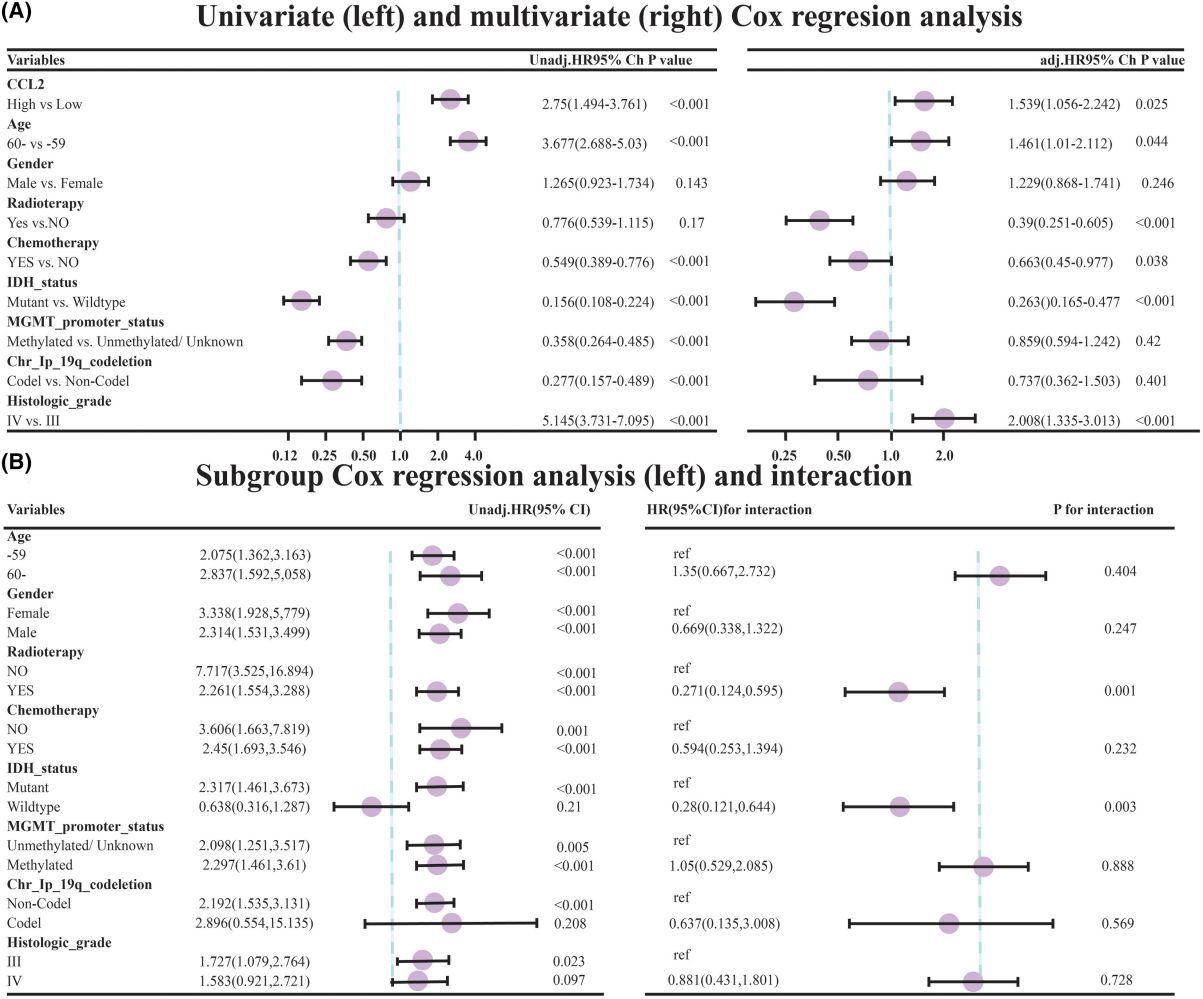
(A) COX regression analyses for each parameter were obtained and summarized, (B) the effects of CCL2 expression level on prognosis of cases in different subgroups of each covariate, CCL2 expression level with other covariates was analyzed by likelihood‐ratio test.

Through the COX proportional‐hazards model, the correlation of one or more factors with the survival outcomes was figured out, facilitating the assessment of the role of the above‐mentioned risk factors. Univariate COX regression was employed to assess the risk factors of OS. Multivariate COX regression was employed for the purpose of determining independent risk factors for OS. The hazard ratio (HR) was used as a metric for making comparison. When the HR value of a factor was >1, this independent variable was taken as a risk factor into account and vice versa. Using the “survival” and “forestplot” R packages, the results of univariate (left) and adjusted multivariate (right) COX regression analyses for each parameter were obtained and summarized in Figure 2A. According to Figure 2A, it was found that the high CCL2 expression level was a risk factor for OS, as revealed by univariate analysis (HR = 2.705, 95% confidence interval (CI): 1.946–3.761, *p* < 0.001). In multivariate analysis, the high CCL2 expression level (HR = 1.539, 95% CI: 1.056–2.242, *p* = 0.025) was a risk factor for OS after the adjustment of cofounders. Furthermore, univariate COX regression was performed to analyze the effects of CCL2 expression level (low CCL2 expression group vs. high CCL2 expression group) on prognosis of cases in different subgroups of each covariate, as shown in the left side of Figure 2B. The interaction of CCL2 expression level with other covariates was analyzed by likelihood‐ratio test, and the results were summarized in the right side of Figure 2B. According to the results of the subgroup analysis, the increased CCL2 expression level was noted as a risk factor for OS in the <60‐year‐old group (HR = 2.075, 95% CI 1.362–3.163, *p* < 0.001). In the ≥60‐year‐old group, the elevated CCL2 expression level was also found as a risk factor for OS (HR = 2.837, 95% CI 1.592–5.085, *p* < 0.001). The *p*‐value of the interaction test between age and CCL expression level was 0.404, and it was not statistically significant. Therefore, no significant correlation was found between CCL2 expression level and age, and the effect of CCL2 expression level on OS was similar between the two age‐based groups.

### 3.3 Correlation analysis of CCL2 expression level with clinical and immunological features of patients with gliomas

**FIGURE 3:**
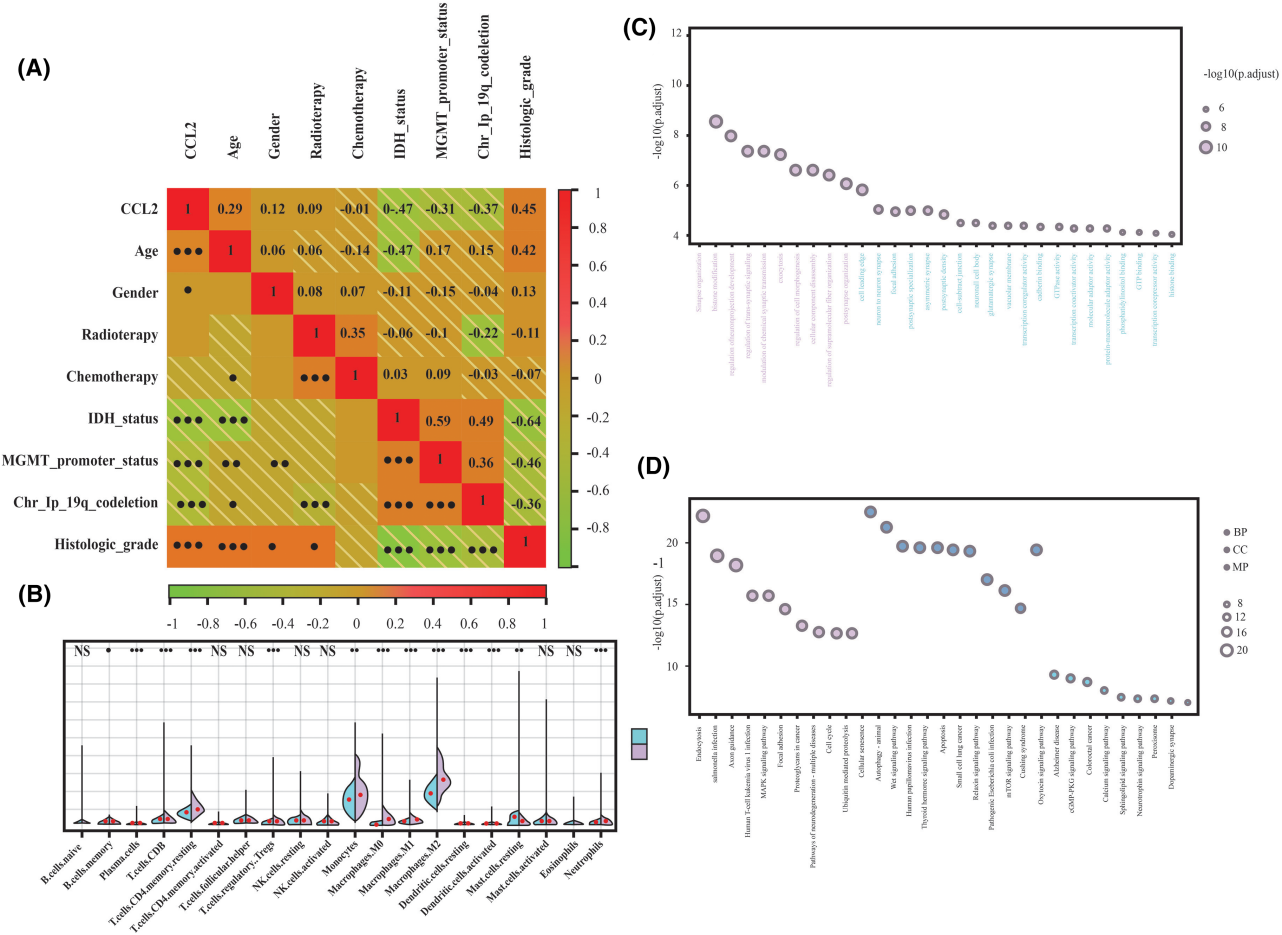
Correlation of CCL expression level with (A) clinical features and (B) immune cell infiltration. ***p < 0.001; **p < 0.01; *p < 0.05; ns: *p* ≥ 0.05. (C) Gene ontology (GO) enrichment analysis results showing top 10 significantly enriched pathways for biological process, cellular component, and molecular function. (D) The top 30 significantly enriched pathways from the results of the KEGG pathway enrichment analysis.

The first 20 features selected through the mRMR method were intersected with the top 20 features selected by the RFE algorithm, and five features were ultimately extracted and selected for the model development using machine learning. The correlation between the host variable (CCL2 expression level) and the clinical features of patients with gliomas was analyzed using the Spearman's correlation coefficient, and the results were shown by a heat map in Figure 3A. The heat map demonstrated a significant correlation of the host variable (CCL2 expression level) with the IDH status, MGMT‐PM status, Chr 1p/19q co‐deletion status, and the histological grade of the tumors (*p* < 0.001).

By uploading the gene expression matrix of glioma samples onto the CIBERSORTx database, it was attempted to calculate immune cell infiltration for each sample. The differences in the degree of immune cell infiltration between the high CCL2 expression and low CCL2 expression groups were displayed by violin plots in Figure 3B. Immune cell infiltration in HGGs was analyzed, and enhanced M2 macrophage infiltration was found in the high CCL2 expression group (*p* < 0.001), whereas no significant difference in the degree of invasion of naive B cells was noted between the two groups.

Furthermore, the CCL2 expression level was also correlated with the genes and pathways identified by enrichment analysis. The GO enrichment analysis indicated enriched differentially expressed genes (DEGs) in the low and high CCL2 expression groups in transcriptional coactivator activity and GTPase activity (Figure 3C). The KEGG pathway analysis indicated that the DEGs in the high and low CCL2 expression groups were significantly enriched in the mTOR signaling pathway,^36^ cGMP‐PKG signaling pathway, and MAPK signaling pathway^37^ (Figure 3D).

### 3.4 Prediction of CCL2 expression level by the SVM‐based radiomics and LR‐based radiomics models

**FIGURE 4:**
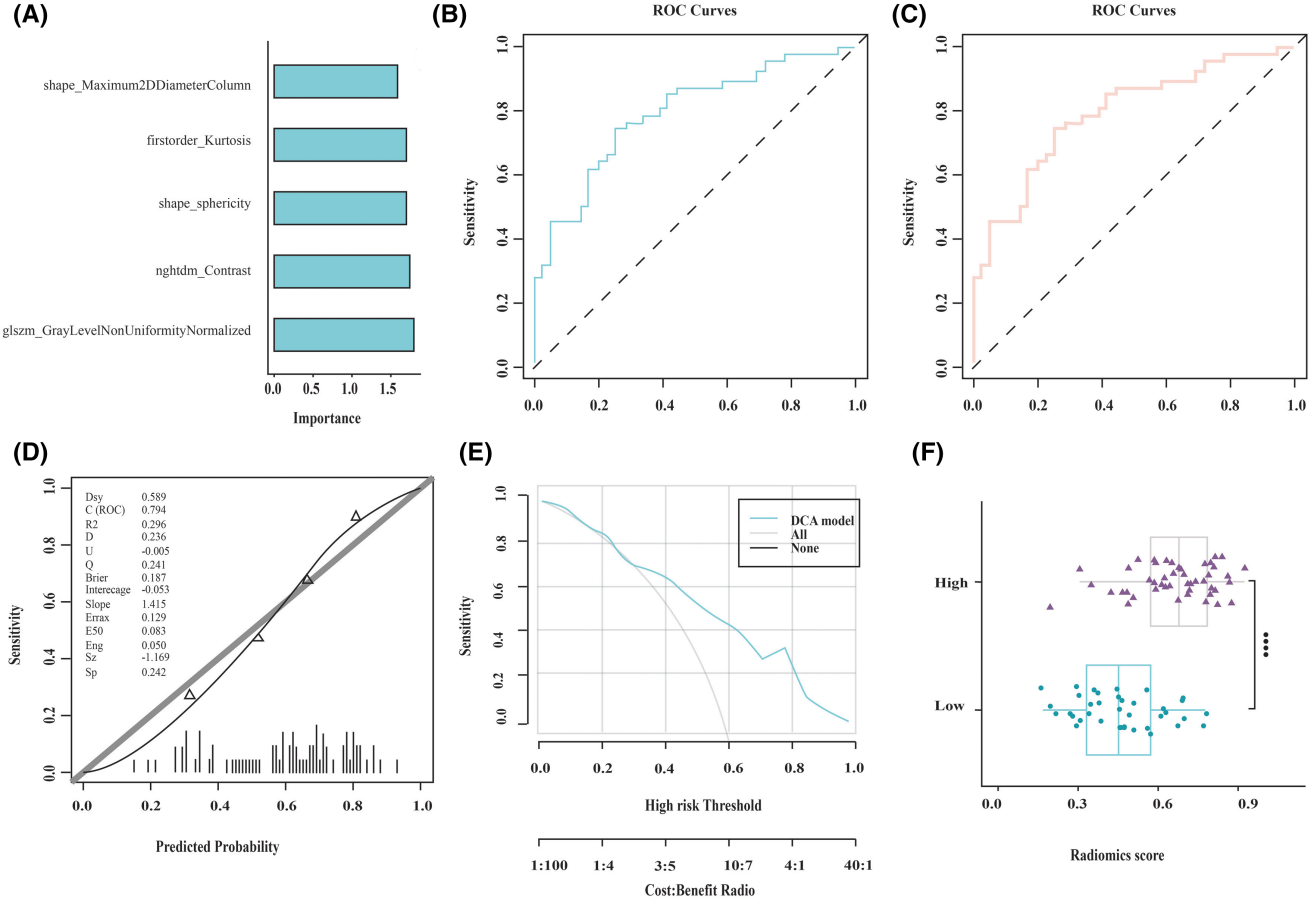
Evaluation of the prediction performance of the SVM‐based radiomics model (A) The significance of the selected features in the establishment of the SVM‐based radiomics model (B) ROC curve of the SVM‐based radiomics model and (C) the ten‐fold cross validation curve of the SVM‐based radiomics model. (D) CCL2 expression level prediction indicated by HLGOF test. (E) DCA showed that the model has satisfactory benefits. (F) The correlation of CCL2 expression level with the SVM‐based Radiomics score.

The SVM‐based radiomics model was developed using 5 features selected by the screening process. The significance of the selected features is presented in Figure 4A. The radiomics model showed a promising predictive performance. As illustrated by the ROC curve in Figure 4B, the area under curve (AUC) value of the model was 0.794, and the AUC of 10‐fold cross‐validation was 0.750 (Figure 4C). The calibration curve and the HLGOF test showed that the radiomics prediction model had a great agreement with the true value (*p* > 0.05) of the prediction in the high CCL2 expression group (Figure 4D). Additionally, DCA (Figure 4E) revealed that the model had a noticeable clinical application. Using the SVM‐based radiomics model, the CCL2 expression level was predicted by the radiomics model (Rad_score). The Rad_score was significantly different between the two groups (*p* < 0.001). The high CCL2 expression group had a higher Rad_score, as shown in Figure 4F.

**FIGURE 5:**
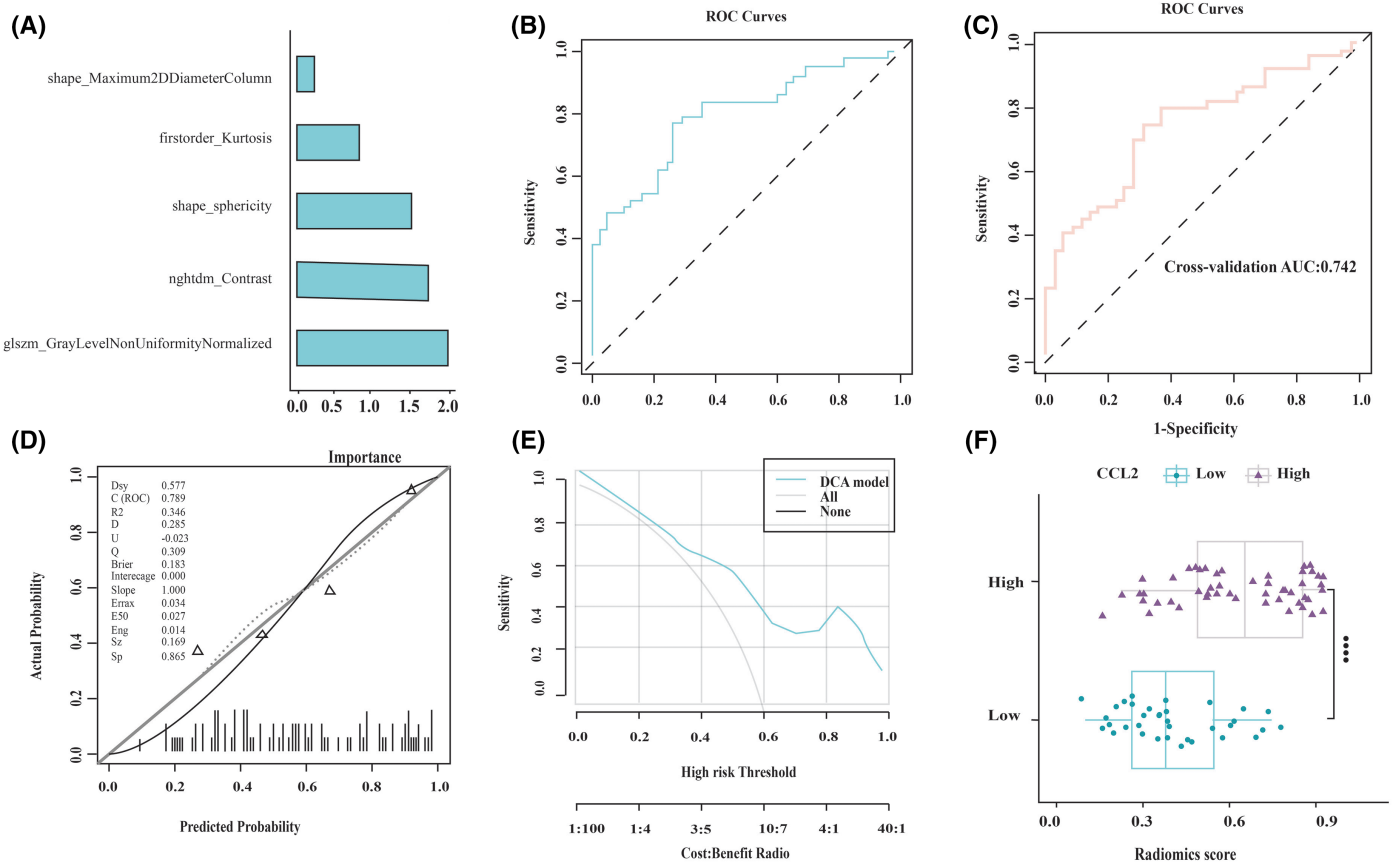
LR model evaluation and prediction (A) The importance of the selected features in the establishment of LR‐based radiomics model (B) ROC curve of the LR‐based radiomics model and (C) the ten‐fold cross validation curve of the LR‐based radiomics model. (D) HLGOF test for the prediction of CCL2 expression level. (E) DCA showed that the model has great benefits. (F) The correlation of CCL2 expression level with the LR‐based Radiomics score.

Additionally, the five selected features were also used to develop a LR‐based radiomics model, in order to compare its performance with that of the SVM‐based radiomics model. And the significance of the five selected features is shown in Figure 5A. The LR‐based radiomics model also exhibited outstanding prediction results. As shown by the ROC curve in Figure 5B, the AUC value of the model was 0.788, and the AUC value of 10‐fold cross‐validation was 0.742 (Figure 5C). The calibration curve and the HLGOF test (Figure 5D) indicated that the prediction performance of the radiomics model was highly in agreement with the true value (*p* > 0.05). Furthermore, DCA (Figure 5E) revealed that the model showed clinical benefits. The AUC, ACC, and other indices of the SVM‐based radiomics model were slightly higher than those of the LR‐based radiomics model; therefore, the prediction results of the SVM‐based radiomics model were used for subsequent analysis. According to the results of DeLong's test, it was found that there was no significant difference between the two groups (*p* = 0.689 and 0.918 for the training and cross‐validation datasets, respectively).

Using the LR‐based radiomics model, Rad_score was also calculated and its correlation with the CCL2 expression level was confirmed, as shown in Figure 5F. Wilcoxon's test compared differences in Rad_score between the two groups. Rad_scores were significantly different between the two groups (*p* < 0.001). Similar to the SVM‐based radiomics model, the higher the CCL2 expression, the greater the Rad_score value.

**FIGURE 6:**
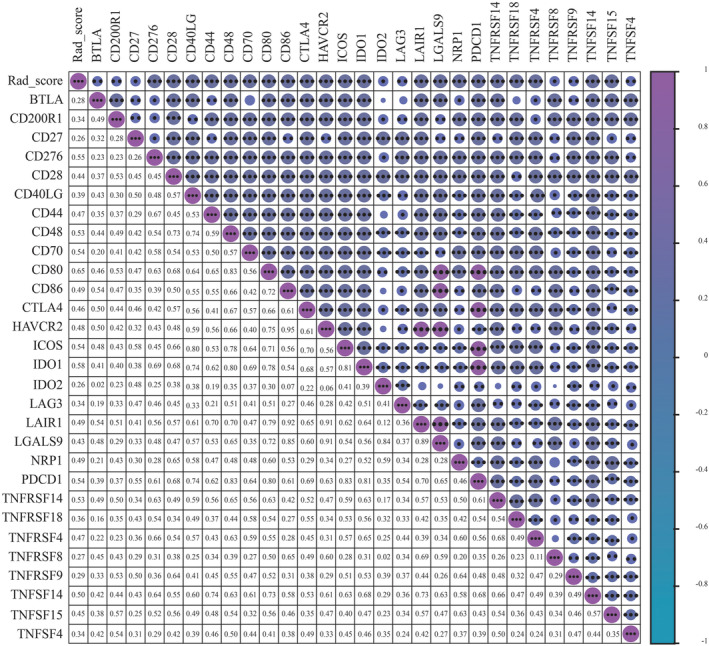
Correlation analysis of Rad_score with immune‐related genes. Selected genes with a significant correlation were indicated.

Additionally, correlation analysis of Rad_score with immune‐related genes^38^ was conducted, and selected genes with a significant correlation (*p* < 0.05) were illustrated in Figure 6. Importantly, Rad_score was positively correlated with glioma‐related genes, such as PDCD1,^39^ CD27,^40^ and CD70.^41^

### 3.5 Radiomics score for survival analysis

To further demonstrate the feasibility and clinical value of using radiomics scores for prognosis prediction of cases with gliomas, the survival analysis of TCIA‐TCGA data was performed using Rad_score. In total, we enrolled 174 cases with HGGs from TCGA database for survival analysis, and they were classified into low Rad_score group (*n* = 69) and high Rad_score group (*n* = 105) with a cutoff value of 0.564. Efforts were made to avoid bias in selecting the 174 cases of HGG used to validate the utility of the SVM‐derived radiomics score. To avoid bias in selecting the 174 cases of HGG used to validate the utility of the SVM‐derived radiomics score, patient selection for validation was performed by more than one person, and the persons involved in patient selection were blinded to the SVM‐derived radiomics score. Patient clinical data were shown in Table 2. There was no significant difference in the sex distribution between the two Rad_score groups (*p* > 0.05), while significant differences were found in the IDH_status, MGMT_promoter_status, Chr_1p_19q_codeletion, and Histologic_grade between the two groups (*p* < 0.01).

**TABLE 2:** Summary of the clinical data of the high Rad_score and low Rad_score groups.

Variables	Total (*n* = 174)	Low (*n* = 69)	High (*n* = 105)	*p*
Age, *n* (%)				1
~59	105 (100)	50 (100)	55 (100)	
Gender, *n* (%)				0.293
Female	76 (44)	34 (49)	42 (40)	
Male	98 (56)	35 (51)	63 (60)	
Radiotherapy, *n* (%)				1
No	27 (16)	11 (16)	16 (15)	
Yes	147 (84)	58 (84)	89 (85)	
IDH status, *n* (%)				<0.001
Wild type	131 (75)	38 (55)	93 (89)	
Mutant	43 (25)	31 (45)	12 (11)	
MGMT promoter status, *n* (%)				<0.001
Unmethylated/Unknown	92 (53)	25 (36)	67 (64)	
Methylated	82 (47)	44 (64)	38 (36)	
Chemotherapy, *n* (%)				0.634
No	32 (18)	11 (16)	21 (20	
Yes	142 (82)	58 (84)	84 (80)	
Chr lp 19q codeletion, *n* (%)				<0.001
Non‐codel	158 (91)	55 (80)	103 (98)	
Codel	16 (9)	14 (20)	2 (2)	
Histologic grade, *n* (%)				<0.001
G3	58 (33)	41 (59)	17 (16)	
G4	116 (67)	28 (41)	88 (84)	
OS, *n* (6)				0.012
Alive	44 (25)	25 (36)	19 (18)	
Dead	130 (75)	44 (64)	86 (82)	
OS, Median (Q1, Q3)	16.25 (8.22, 25.98)	19.5 (7.13, 39.63)	14.6 (8.47, 23.03)	0.024

**FIGURE 7:**
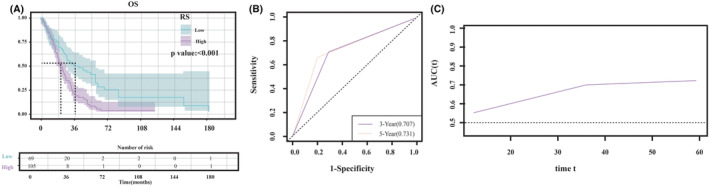
(A) KMSC for patients in the high and low Rad_score groups. (B) The time‐dependent ROC curves for the radiomics model for predicting the overall survival. (C) AUC curves for HGG patients at different time points.

Through the “survival” R package, it was attempted to assess the variations of survival rates among different groups via the KMSC, where the survival rate of 50% was indicative of median survival time. As illustrated in Figure 7A, the median survival time was 16.3 months in the high Rad_score group and 27.3 months in the low Rad_score group. The significant association of the high Rad_score with the reduced OS rate was confirmed via the KMSC (*p* < 0.001).

In order to carry out survival analysis, disease states and influential factors may change over time. Therefore, time‐dependent ROC curves were drawn according to different time points to assess the prediction ability of factors time dependently. The corresponding time‐dependent ROC curves were drawn at 3 and 5 years after diagnosing HGGs (Figure 7B), and the AUC values were calculated for the purpose of comparing differences between the two groups (Rad_score vs. survival) at various time points (Figure 7C). The AUC values for Rad_score on the predictive prognostic ability were 0.707 and 0.731 for 3‐ and 5‐year OS, respectively, and the AUC values increased over time.

## 4 DISCUSSION

Gliomas are the most prevalent CNS tumor. While CCL2 is a proven good marker that could define the progress and malignancy of gliomas accurately, its CSF level is an invasive procedure.^42^ That causes a limit on the frequency that CCL2 expression level can be acquired. A model that linked MRI images with CCL2 expression level reduces cost and invasive procedures, and enables oncologists to analyze treatment effects, and re‐stage and adjust treatment plans more accurately. Therefore, the new model may improve patients' prognosis.

Notably, this is the first comprehensive study that used MRI‐based radiomics to predict CCL2 expression levels in HGG patients. It was revealed that the CCL2 expression level is a risk factor for OS using the KMSC. The COX regression analysis showed that CCL2, as a single risk factor, could affect the clinical outcome regardless of patients' age.

The radiomics has noticeably attracted oncologists' attention due to its ability in providing valuable diagnostic and prognostic data. Additionally, in order to develop more precise prognostic and therapeutic models, numerous methods on the basis of artificial intelligence, for example, machine learning and deep learning, were developed to reveal whether clinical symptoms would be correlated with genetic characteristics.^43^ Regarding machine learning, the development of a robust radiomics‐based algorithm is feasible using data exclusiveness. Importantly, MR and CT images, protein sequences, and genetic patterns are valuable for extracting numerous features on the basis of a feature extraction strategy.^44^, ^45^ These features should be subsequently imported into the machine learning algorithms, while different contributions of different features to predictive models are noteworthy. Therefore, the results may be remarkably affected by selecting appropriate features. Various methods were proposed to select appropriate features. For instance, Le et al.^46^ employed the Spearman's correlation test and F‐score analysis to select important features for glioblastoma detection. Using multivariable logistic regression analysis, Kha et al.^47^ explored a radiomics signature for prediction of 1p/19q co‐deletion status. However, most of these studies were simplistic rather than clinical, omitting the correlation between selected features and the patients' outcome.

In the present study, the KMSC was plotted to illustrate the variations of survival rates in diverse groups. And a median survival of 44.63 months was achieved in the low CCL2 expression group, while it was only 16.83 months in the high CCL2 expression group. The significant association of the higher CCL2 expression level with the reduced OS rate was confirmed by KMSC. It was also indicated that age older than or equal to 60 years was significantly associated with the reduced OS rate. Thus, highly expressed CCL2 was confirmed, and it was identified as a significant risk factor for OS rate in patients with gliomas. Lundemann et al.^48^ developed a voxel‐based prediction model (AUC, 0.77) for the purpose of predicting the recurrence region of glioma via positron emission tomography (PET), diffusion‐weighted imaging (DWI), and dynamic contrast‐enhanced imaging. In another study,^49^ models were developed for predicting OS of HGG cases using analyzing contrast‐enhanced T1‐weighted (CE‐TIW) and fluid‐attenuated inversion recovery (FLAIR) images, in which concordance index (C‐index) was equal to 0.76. Liu et al.^50^ similarly attempted to analyze T2W images for low‐grade gliomas and they could obtain a C‐index of 0.82. The AUC and C‐index may reflect the performance of prediction models, in which AUC is correlated with the C‐index on the binary classification task, and they may be both compared to a certain extent.

The subgroup analysis revealed that the increased CCL2 expression level was noted as a risk factor for OS in the <60‐year‐old and ≥ 60‐year‐old groups. As a result, no significant association was found between CCL2 expression level and age, and the effect of CCL2 expression level on OS was similar between the two age‐based groups. According to the Spearman's correlation analysis, a significant correlation of the host variable (CCL2 expression level) with the IDH status, MGMT‐PM status, Chr 1p/19q co‐deletion status, and the histological grade of the tumors was identified via the heat map. In addition, immune cell infiltration in HGGs was analyzed, and enhanced M2 macrophage infiltration was found in the high CCL2 expression group, whereas no significant difference in the degree of invasion of naive B cells was noted between the two groups. The presence and activity of macrophages, particularly M2 subtype, play a crucial role in the recurrent GBM.^51^, ^52^, ^53^, ^54^ In the recurrent GBM, the tumor microenvironment undergoes significant changes, with increased infiltration of various immune cells, including macrophages.^55^ M2 macrophages may exhibit immunosuppressive and pro‐tumoral functions, promoting tumor progression and therapeutic resistance.^56^, ^57^, ^58^ They contribute to the formation of an immunosuppressive microenvironment in the tumor, developing tumor growth, invasion, and angiogenesis. Moreover, M2 macrophages can reduce anti‐tumor immune responses, impairing the efficacy of immunotherapies and conventional treatments.^55^ Therefore, the finding of enhanced M2 macrophage infiltration in the high CCL2 expression group suggested a potential mechanism by which CCL2 may contribute to tumor progression and therapeutic resistance in the recurrent GBM. Targeting the CCL2‐M2 macrophage axis could represent a promising therapeutic strategy to improve outcomes in patients with recurrent GBM, potentially by reversing immunosuppression and enhancing antitumor immune responses.

The GO enrichment analysis showed the significant enrichment of DEGs in the high and low CCL2 expression groups in transcriptional coactivator activity and GTPase activity. The KEGG pathway analysis indicated the significant enrichment of DEGs in the low and high CCL2 expression groups in the mTOR signaling pathway, cGMP‐PKG signaling pathway, and MAPK signaling pathway. According to the calibration curve and the HLGOF test, a great agreement of the radiomics prediction model with the true value of the prediction in the high CCL2 expression group was confirmed. Additionally, DCA indicated that the model had a noticeable clinical application. Using the SVM‐based radiomics model, the CCL2 expression level was predicted by the radiomics model (Rad_score). The differences in Rad_score were compared by the Wilcoxon test between the low and high CCL2 expression groups. The Rad_score significantly differed between the two groups. Using the LR‐based radiomics model, Rad_score was also calculated and its correlation with the CCL2 expression level was confirmed. Similar to the SVM‐based radiomics model, the higher the CCL2 expression, the greater the Rad_score value. Rad_score was positively correlated with glioma‐related genes, such as PDCD1, CD27, and CD70.

However, this study had some limitations. First, the number of physicians for delineating VOIs needed to be increased to further include/exclude features for developing machine learning models. Second, the study only concentrated on HGGs, and assessment of low‐grade gliomas may provide new insights into the model performance and expand it for a wider range of applications. Lastly, the prediction of CCL2 expression level was mainly targeted, while other risk factors, including IDH mutation and MGMT‐PM would be highly correlated with the CCL2 expression level. It is suggested to indicate whether the proposed model can predict the functions of other risk factors to understand molecular imaging. Future research may involve more cases and recruit more physicians for delineating VOIs and marking other characteristics to facilitate machine learning. Additional risk factors and molecular targets may be investigated. New advances in the fields of radiomics and machine learning may be applied to the model as well. For instance, SHapley Additive exPlanations (SHAP) may make the output of the model more understandable.^59^

## 5 CONCLUSIONS

In conclusion, this study provided comprehensive insights into the prognostic value of CCL2 expression level in HGGs through integrated analysis of transcriptomic data from TCGA and imaging data from TCIA. It was demonstrated that a high CCL2 expression was significantly associated with the reduced OS rate in HGG patients, independent of age and other clinical factors. Additionally, the MRI‐based radiomics models exhibited promising performance in predicting CCL2 expression level, suggesting a noninvasive approach to assess molecular characteristics of tumors. Furthermore, the analysis revealed correlations between CCL2 expression and clinical features, immune cell infiltration patterns, and enrichment of specific pathways, highlighting the complex correlation between CCL2 signaling and glioma progression. While this study highlighted the potential of radiomics in bridging imaging and molecular data for clinical decision‐making, further large‐scale research is warranted to comprehensively validate the utility of the SVM‐derived radiomics signature in real‐world clinical settings, confirming its predictive accuracy and assessing its impact on patient outcomes. Future directions may involve refining machine learning algorithms, incorporating advanced imaging techniques, and prospectively evaluating the clinical utility of radiomics in personalized treatment strategies for glioma patients.

## AUTHOR CONTRIBUTIONS

**Qingqing Zhou:** Data curation (equal); methodology (equal); writing – original draft (equal). **Yamei Wang:** Conceptualization (equal); formal analysis (equal); resources (equal); writing – original draft (equal). **Qing Zhang:** Data curation (equal); resources (equal); software (equal); supervision (equal); validation (equal). **XiaoMing Wei:** Formal analysis (equal); investigation (equal); methodology (equal); validation (equal); visualization (equal). **Yuan Yao:** Project administration (equal); writing – review and editing (equal). **Liang Xia:** Funding acquisition (equal); methodology (equal); project administration (equal); writing – review and editing (equal).

## FUNDING INFORMATION

This study was supported by Natural Science Foundation of Zhejiang Province (Grant No.: Y22H167258, LY21H160007), and Zhejiang Medical Science and Technology Project (Grant No.: 2022RC16).

## CONFLICT OF INTEREST STATEMENT

The authors declare that there is no conflict of interest.

## ETHICS APPROVAL AND CONSENT TO PARTICIPATE

The Ethics Committee the First Affiliated Hospital of Yangtze University approved the study protocol.

## Data Availability

The authors confirm that the data supporting the findings of this study are available within the article.
